# Health and well-being of refugees, asylum seekers, undocumented migrants, and internally displaced persons under COVID-19: a scoping review

**DOI:** 10.3389/fpubh.2023.1145002

**Published:** 2023-04-26

**Authors:** Rabie Adel El Arab, Joel Somerville, Fuad H. Abuadas, Esther Rubinat-Arnaldo, Mette Sagbakken

**Affiliations:** ^1^Faculty of Nursing and Physiotherapy, University of Lleida, Lleida, Spain; ^2^Institute for Biomedical Research (IRBLleida), Healthcare Research Group (GRECS), Lleida, Spain; ^3^Department of Optometry, Inverness College, University of the Highlands and Island, Inverness, United Kingdom; ^4^Community Health Nursing Department, College of Nursing, Jouf University, Sakaka, Saudi Arabia; ^5^Faculty of Health Sciences, Department of Nursing and Health Promotion, Oslo Metropolitan University, Oslo, Norway

**Keywords:** refugees, asylum seekers, COVID-19, vaccination, health care, undocumented migrants, internally displaced persons

## Abstract

**Objectives:**

The objective of this scoping review was to identify what is known about the impact of COVID-19 on the physical and mental well-being of refugees, asylum seekers, undocumented migrants, and internally displaced persons. The aim was also to identify barriers influencing access to treatment or prevention.

**Methods:**

The search was conducted using PubMed/Medline, CINAHL, Scopus, and ScienceDirect. A mixed methods appraisal tool was used to assess methodological rigor. The study findings were synthesized using a thematic analysis approach.

**Results and Discussion:**

This review comprised 24 studies and were conducted utilizing a mixed method approach incorporating both quantitative and qualitative methodologies. Two major themes were identified related to the impact of COVID-19 on the health and wellbeing of refugees, asylum seekers, undocumented migrants, and internally displaced persons and the key barriers influencing access to treatment or prevention of COVID-19. They often have barriers to accessing healthcare due to their legal status, language barriers, and limited resources. The pandemic has further strained already limited health resources, making it even more challenging for these populations to receive healthcare. This review reveals that refugees and asylum seekers in receiving facilities face a higher risk of COVID-19 infection than the general population due to their less favorable living conditions. The various health impacts stem from a lack of access to accurate information about the pandemic, misinformation, and the exacerbation of pre-existing mental health issues caused by heightened stress, anxiety, and uncertainty, fear of deportation among undocumented migrants, and overcrowding camps and detention facilities that increase exposure risk. Social distancing measures are difficult to implement in these settings, and inadequate sanitation, hygiene, and a lack of personal protective equipment further compound the problem. Moreover, the pandemic has had significant economic consequences for these populations. Many of them rely on informal or precarious employment, which has been disproportionately affected by the pandemic. Job losses and reduced working hours, and limited access to social protection can lead to increased poverty, and food insecurity. Children faced specific challenges, such as disruptions to education, additionally, interruptions in support services for pregnant women. Some pregnant women have avoided seeking maternity care due to fears of contracting COVID-19, resulting in increased home births and delays in accessing healthcare services. Factors that play a role in vaccination reluctance include uncertainty of undocumented migrants’ inclusion in vaccination programs, furthermore, a growing vaccine hesitancy in the population; skepticism about the safety of vaccines, inadequate knowledge/education, a variety of access barriers such as language barriers, and logistical challenges including remote locations, and inaccurate information.

**Conclusion:**

This review highlights that the physical health of refugees, asylum seekers, undocumented migrants, and internally displaced persons has been significantly impacted by various barriers to healthcare access during the pandemic. These barriers include legal and administrative challenges, such as a lack of documentation. Additionally, the shift to digital tools has introduced new obstacles, not only due to language barriers or limited technical knowledge but also because of structural barriers, such as the requirement of a bank ID that is often inaccessible to these groups. Other factors contributing to limited healthcare access include financial constraints, language barriers, and discrimination. Additionally, limited access to accurate information about health services, prevention measures, and available resources may hinder them from seeking care or following public health guidelines. Misinformation and lack of trust in healthcare systems can also contribute to a reluctance to access care or vaccination programs. There is concerning evidence regarding vaccine hesitancy that needs to be addressed to reduce any future pandemic outbreak, in addition there is a need to explore the factors that play a role in vaccination reluctance among children in these populations.

## Background

The number of refugees and other displaced individuals in mid of 2022 was estimated to be approximately 103 million (1). Many migrants, asylum seekers and refugees find their final destination is not what they expected. They may spend months or years in conditions frequently fall short of basic humanitarian standards even in the European Union (EU) (2, 3). The legal and socio-economic status varies greatly; however, many are at a higher risk of inadequate healthcare due to their unsettled or transitory status (4). The European Centre for Disease Prevention and Control (ECDC) has reported that migrants in Europe have disproportionately been affected by the Coronavirus disease (COVID-19) pandemic in terms of health and social impacts (5). Thus, COVID-19 has exposed and exacerbated many preexisting health inequalities within communities and between populations (6). Due to overcrowding, lack of access to clean water and sanitation, and inadequate medical care, there is a high risk of transmission of the SARS-Cov-2 virus (7). Furthermore, certain migrant groups have several factors contributing to low vaccine uptake and may experience access barriers (5). Adult and adolescent migrants may not receive the recommended regular vaccinations and are not immediately included in catch-up vaccination efforts in various European countries (8). The COVID-19 pandemic may increase the vulnerability of refugees and asylum seekers, and the lack of COVID-19 health information strategies for culturally and linguistically diverse groups reduces awareness of prevention measures (2, 3). A variety of risk factors and comorbidities associated with COVID-19 have exacerbated health disparities and contributed to the increase of disease burden (9). According to Kabir et al. (10), the government’s goals and decisions during the epidemic prioritized its inhabitants, putting the population of refugees at serious risk. Most nations have closed their borders in an effort to stem the spread of COVID-19, and this has allowed them to take legally dubious, stern steps toward refugees and migrants (11). In the absence of official status in a host country, migrants often live on the fringes of society without adequate access to basic needs (12). In order to enhance migrants’ welfare and immunization programs and tactics, including the COVID-19 vaccination, it is crucial to study how COVID 19 affects migrants’ physical and mental health as well as the obstacles that prevent certain migrants from getting the vaccination they need.

The following research questions directed the review of the literature:

What is known about how COVID-19 affects the physical and mental health of refugees, asylum seekers, undocumented immigrants, and internally displaced persons?

What is known about the barriers influencing access to treatment or prevention of refugees, asylum seekers, undocumented immigrants, and internally displaced persons during the COVID-19 pandemic?

## Methodology

The scattered and fragmented nature of the literature made conducting a systematic review impossible. The subject is also comparatively understudied and cuts across several approaches and disciplines. The aim of this study is to map the current research on this subject that has been done using scholarly publications, including qualitative, quantitative, and mixed methodologies studies. The criteria used for inclusion and exclusion of studies are listed in Table 1. In view of the nature of the task and the research’s current state, a scoping review was considered appropriate (13). The study follows the PRISMA 2020 guidelines for scoping reviews (14). The search was conducted using PubMed/Medline, CINAHL, Scopus, and ScienceDirect, and the key search terms were defined using a PEO (population, exposure, outcome) strategy (15). The following PEO components were used during the search:

**Table 1:** Inclusion and exclusion criteria.

Inclusion	Exclusion
Studies that focus on refugees, asylum seekers, and undocumented immigrants, internally displaced persons of all ages in low-, middle-and high-income countries. Studies that report health-related information. Studies that focus on drivers, barriers, or challenges. Peer-reviewed articles. Primary research articles (qualitative, quantitative of mixed methods research). Articles published from December 2019 to October 2022.	Studies not published in English. Non-original research articles (e.g., abstracts, conference papers, reviews, commentaries, editorials, reports, grey literature).

Population: Refugees, asylum seekers, undocumented migrants, and internally displaced persons.

Exposure: COVID-19 infection or vaccination.

Outcome: Health and well-being, and barriers.

**Figure1:**
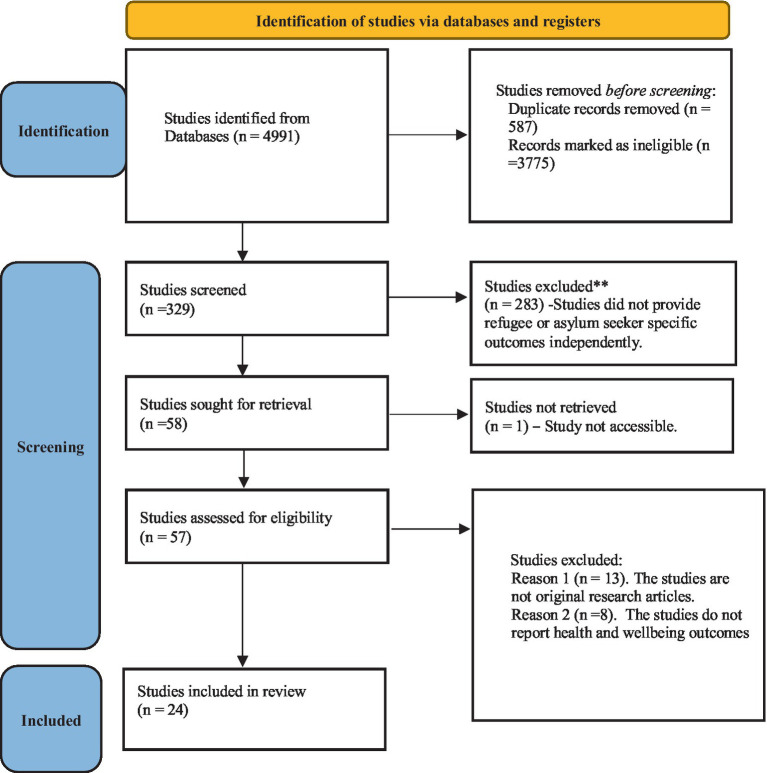
PRISMA Flow Chart.

To manage references, look for duplicates, and eliminate them, EndNote 9.2 (16), was used as a citation management tool. In order to keep the precise numbers retrieved from each database, the references were exported to the software. The screening was conducted using the Rayyan tool (17), and the references were exported to Rayyan after deduplication. According to the inclusion and exclusion criteria, two reviewers (RA and JS) independently examined the references’ titles and abstracts, and a third reviewer settled any disagreements (FA). We looked for pertinent references in the complete texts of the articles. The title/abstract screening step for the full-text publications was conducted using the same methods as in the title/abstract screening. Author, title, publication year, the country where the study was performed, study design, and sample were gathered from the publications using a customized data charting form recommended by Arksey and O’Malley (2007) (18). The four databases yielded a total of 4,991 articles. Following the removal of duplicates and title scanning, 329 articles were selected for screening. Additionally, 271 studies were disregarded because they failed to present results particular to refugees or asylum seekers independently. Of the 58 articles which were retrieved fully, only 24 met all the inclusion and exclusion criteria (Figure 1). The articles included were coded thematically and classified according to major themes and subthemes (19). In this review, the terms of migrants, asylum seekers, refugees, internally displaced persons, and undocumented migrants are used as adopted by the International Organization of Migration (Appendix I) (20).

Researchers can use critical analysis to focus their attention on the articles that are most pertinent to the research issue and that can substantiate their assertions with reliable evidence, or to steer them toward high-level research that is directly useful to their practice (21). In the current study, the mixed methods appraisal tool (MMAT) was utilized, see (Appendix II). This tool makes evaluating qualitative, quantitative, and mixed-methods research easier than alternative tools (22).

## Results

Study findings are broadly categorized into two major research themes: the impact of COVID-19 on asylum seekers, refugees, internally displaced persons, and undocumented migrants’ health and well-being, and the key barriers to COVID-19 treatment and prophylaxis.

Studies were conducted in a variety of locations. In total, 13 studies were carried out in high-income countries (23–35), and 11 in low-to middle-income countries (36–46). There is a diversity of research designs in the studies that were included. There are a total of five qualitative research studies (23, 31, 33, 41, 45), five mixed methods studies (34, 36, 38, 42, 46), while 14 studies adopted a quantitative research design (21, 23–26, 29, 32, 34, 37, 40, 41). See summary table (Appendix III).

### Health and wellbeing of asylum seekers, refugees, internally displaced persons, and undocumented migrants under COVID-19

#### Prevalence of COVID-19 in the target population

The prevalence of COVID-19 among the target population has been reported in four studies (24, 26, 30, 35). Turunen et al. (26) report that despite prior preventative steps being implemented, the number of asylum seekers presenting with COVID-19 in April 2020 at a receiving facility in Espoo, Finland, rose. In the screening of the entire population, 37 percent (n = 260) tested positive and were isolated. The local public health authority quarantined the other asylum seekers who tested negative for 14 days to prevent further spread. After the quarantine was lifted, no widespread transmission of COVID-19 was detected, suggesting that comprehensive quarantine and isolation measures likely contained the outbreak. During the first 9 months of the epidemic in Greece (26th February–15th November 2020), a retrospective analysis of national surveillance data was carried out to detect COVID-19 outbreaks and estimate incidence among asylum seekers and refugees residing in the camps (first wave, second wave, and overall, over the entire nine-month period) (30) There were 25 COVID-19 outbreaks found in refugee and asylum seeker institutions. The study’s findings showed that, due to their less favorable living conditions, refugees and asylum seekers in receiving facilities had a 2.5–3 times greater risk of COVID-19 infection than the general population (30).

#### Economic factors influencing health and overall wellbeing

Some studies recognized an indirect impact of COVID-19 on asylum seekers, refugees, internally displaced persons, and undocumented migrants’ overall wellbeing (24, 36, 41). In a study from Burkina Faso, for instance, the authors compared the living conditions of IDPs before and after the authorities imposed a lockdown. They found that 85% of the IDPs surveyed had no income-generating activities during the lockdown, and the remaining 15% who continued to work reported that their activities had been significantly scaled down. For the vast majority of them, their living situations, which were already considered to be challenging under “normal” circumstances (inadequate food, little financial help, or challenging access to healthcare), continued to deteriorate. IDPs were also prohibited from leaving the camps or areas where they were housed to look for better living circumstances or to go back home (36).

In a study among among US Bhutanese and Burmese refugees (24) comparing those with and without infection with severe acute respiratory syndrome coronavirus, it was found that essential workers (working in areas with high risk of exposure to COVID-19 and limited worker protections), were more likely to contract the disease and spread it to their families. Besides higher exposure and less worker protection, the findings should be considered in light of the fact that refugees are less likely to have access to public and occupational health information in their preferred language. Further, poverty may make people more vulnerable, such as refuges without the means to obtain personal vehicles being dependent on crowded public transport (24).

Similarly, in a qualitative study exploring the challenges of Afghan refugee women in the face of COVID-19 in Iran, Lebni et al. (41) found that challenges related to access to information resources about COVID-19, family challenges (such as intensified experience of violence and conflict in the family), socio-economic challenges (such as exacerbation of economic problems, high-risk living conditions, social isolation, limited support of social and health organizations), health issues (problems related to treatment, injustice in providing services and facilities) all contributed to women being vulnerable to COVID-19 infection.

#### Impact on mental health

According to three studies among refugees and asylum seekers with the aim of assessing the relationship between COVID-19 related fear and stressors and mental health, it was found that depression, anxiety and stress in relation to the fear of being infected, and associated consequences, were moderate to high (27, 28, 37). In one of the studies, among 274 Syrian refuges in Canada (27), severe anxiety (26.8%) depression (12.2%) and stress (9,7%) were associated with the fear of contracting COVID-19. In a study among 656 refugees and asylum seekers living in Australia Liddell et al. (28) found that worries about contracting or spreading the potentially deadly COVID-19 virus were associated with health anxiety and post-traumatic stress disorder (PTSD). In general, fear related to the future, such as worries about the application process, predicted health anxiety. Social difficulties, such as having to stay at home or not being able to engage in social activities were associated with depression. However, the strongest predictor of negative mental health outcomes was related to COVID-19 being a reminder of previous difficult life events (28). Mental health challenges have also been associated with other factors, including a lack of access to resources (37), inadequate health information (28, 37), perceived discrimination by host country actors (28, 37) and poor social support (37).

Studies show that low social support and a lack of knowledge about the steps that needs to be taken to lower the prevalence of stress, depression, and anxiety are related (28, 37). For example, in the study by Sharif-Esfahani et al. (27) there was a positive association between a person’s sense of belonging to Canada and stress and anxiety measures. The lower once sense of belonging to Canada, the higher levels of stress and anxiety – a situation likely to have been exacerbated by isolation and a lack of social support during the pandemic.

#### Impact on specific groups

The prevalence of physical and mental health problems across specific groups, such as adolescents, has been recognized. Jones et al. (38) investigated the extent to which the pandemic has compounded pre-existing social inequalities among adolescents in Jordan and reported that 19.3% of the adolescents in the sample presented with symptoms of moderate-to severe depression 9 months into the pandemic and had small signs of improvement. Additionally, the pandemic had caused significant service and economic disruptions that had a wide range of diverse implications on teenage wellbeing. Gugliemi et al. (42) found that among Rohingya teenagers of both sexes in Bangladesh, the pandemic has increased food insecurity, educational and economic marginalization, threats to one’s bodily integrity, and a decline in reported health status. Ceccon and Moscardino (34) found that young adult asylum seekers in Italy suffered more psychological and physical stress throughout the pandemic. The increased stress was related to worries about family in their home country, fear of ones’ health, and concerns due to delays in processing asylum applications. During the lockdown, many experienced a sense of hopelessness as they were without employment and faced significant uncertainty. Another specific group paid attention to are pregnant women. Due to, among others, concerns about contracting the disease, Lebni et al. (41) noted that Afghan refugee women did not seek support for pregnancy and childbirth. Similarly, a qualitative study from Kenya found that during the pandemic, preferences for home births rose, and healthcare professionals saw delays in seeking care as well as an overall decline in the use of maternal health care services due to a fear of contracting Covid-19 (45).

### Barriers influencing access to treatment or prevention

#### lack of access to information regarding COVID-19

According to different studies, refugees may not have access to the latest and most accurate information regarding the pandemic, and the information are not always provided in a language they can understand (26, 28, 43). Budak and Bostan (40) found that 30 percent of Syrian refugees lacked information about self-protection during the pandemic. This was attributed to limited access to digital tools and difficulty in communicating with healthcare workers. In their study on Afghan women in Iran, Lebni et al. (41) found that a lack of information regarding COVID-19 resulted in a resistance to explore treatment and preventative alternatives. Further, most of the Afghan women were illiterate or had low literacy levels, making it difficult to access various information about COVID-19 (41). In a study from Sweden (20) it was found that the shift from face-to face encounters within health care for refugees to more digital assessment, influenced access to health care among those not fluent in Swedish. The use of translators was found to be more complicated, and the quality of the translation decreased, often due to excessive background noise. Further, the shift to digital tools created new barriers, not only due to language barriers or lack of technical knowledge, but also due to structural barriers such as using such tools requiring a Swedish bank ID, often inaccessible to refugees in Sweden (23).

#### Lack of access to necessary resources

Studies indicate that refugees in a variety of contexts have been prevented from accessing treatment or prevention, e.g., unable or being delayed in getting important medical appointments during the pandemic (23) or experiencing limited access to health services and/or human aid in the form of food, water, and shelter (36). Also, personal protective equipment, such as masks and gloves, are found to be restricted in typical refuge settlements; an important factor in the effort of preventing the spread of COVID-19 among the refugee population (37). According to a modeling study by Truelove et al. (39), ineffective control of the viral spread of COVID-19 through refugee camps or settlements can result in excessive stress on healthcare systems, particularly in a high-population density refugee settlement. Further, in their modeling research, Gilman et al. (22) claim that the situation is made worse by the difficulty to segment camps to separate COVID-19-positive individuals as well as by the restricted availability of face masks. Even though a study from Kenya found that fear of contracting COVID-19 was one of the main reasons for the decline in the use of maternal health care services among refugees, financial obstacles like the inability to afford masks for antenatal visits and a general lack of refugees-inclusive health care were also identified as significant barriers (45).

In a qualitative study among refugee women in California, United States, (33) exploring the receptiveness for virtual platforms to access information and engage in discussions about reproductive health, it was found that literacy, language proficiency, and access to and experience with digital technology highly influenced the women’s engagement and actual possibilities to navigate this type of digital support. The study also identified cultural barriers, including the need for all-female support in virtual settings to preserve confidentiality and modesty (33).

#### Vaccine hesitancy barriers

Studies show that there are several factors that contribute to vaccine hesitancy. The lack of knowledge about how the vaccine will protect the individual and information on its administration is one of the key aspects highlighted (31, 41). According to different studies (32, 41, 44), lack of formal education and lack of trust are important barriers to vaccine hesitancy. Furthermore, three different studies among refugees in Lebanon, Iran, and the USA, show that misinformation spread through social media can interfere with judgments regarding vaccinations (29, 35, 41). It seems like refugees often prefer social media tools because they are more suited to their language and literacy skills and because refugee camps often lack access to organized information. However, due to algorithmic effects, such preferences may generate or worsen vaccination reluctance and lead to serious misconceptions.

Studies show that concerns regarding the effectiveness and safety of the vaccine contribute to vaccine hesitancy. For instance, a survey of 3,173 Syrians in Lebanon (44) found that 31 percent of the refugees did not want the vaccination, and 7 percent were undecided. Concerns about the vaccine being too new and insufficiently tested (24%), the desire for precautionary measures (27%), and the idea that the vaccination is not essential (21%) were given as justifications. Further, studies among refugees living in the United Kingdom and the United States showed a widespread concern about the adverse effects of the vaccine and its implications for safety (31, 32). In a study among 812 undocumented migrants in Geneva, Baltimore, Milano, and Paris (35) it was revealed that 77% of the respondents approved of vaccination generally, but 56% disapproved of the COVID-19 vaccination specifically. Participants mainly searched for information through traditional or social media sources as well as community networks; sources that may have influenced people’s reluctance. For example, getting information through social media in Milano and community networks in Paris were negatively associated with demand for vaccination. Furthermore, participants who believed that COVID-19 vaccination would be unavailable to undocumented migrants cited lack of health insurance or card as the main barrier to access (35).

Other reasons associated with vaccine hesitancy are attributed to confusion over the registration process, language and/or communication barriers, and limited trust in the health care system (31, 32, 41). According to Liddell et al. (29) and Shaw et al. (32) logistical challenges in accessing vaccine centers, such as transportation barriers, also contribute to vaccine hesitancy among refugees. In general, the findings in the above studies indicate a general fear related to the effects of the COVID-19 vaccine, combined with factors such as lack of access, communication barriers, fear of the vaccine being religiously prohibited, and a general lack of trust in health authorities; potentially reflecting prior experiences with persecution and lack of protection by authorities in their home countries.

## Discussion

A pandemic or epidemic may result in disproportionately high mortality rates for marginalized groups, as observed in previous pandemics and epidemics (47). As part of this scoping review, only one study in Greece (30) compared COVID-19 infection rates among refugees and the general population and found a higher prevalence of infection among refugees. An ECDC report (5) indicates that some migrant communities are disproportionately represented in COVID-19 cases, hospitalizations, and deaths. In Denmark, Norway, and Sweden, for example, COVID-19 cases have had higher proportions of migrants than expected given their numbers in population (5).

The modeling studies evaluated in this research (25, 39) indicate that crowded conditions and delays in accessing medical care are additional risk factors. Prior research has shown a lack of access to preventative health support for migrant populations and ethnic minorities, who bear a disproportionately high burden when it comes to infectious diseases (48). It is necessary to conduct more population-based epidemiological studies to evaluate the degree of inequity in access to healthcare between refugees and the general population.

The first research question was to identify the impact of the pandemic on the physical and mental health of asylum seekers, refugees, internally displaced persons, and undocumented migrants. Based on an analysis of the mental health impact of the pandemic, anxiety and depression have been identified as common outcomes (27, 28, 37). However, the reasons for the negative impact on mental health should be investigated further. The researchers in this review argue that refugees are likely to experience trauma due to fleeing war, violence, persecution, and discrimination. They also face additional challenges of stress and fear of contracting COVID-19 or losing their livelihoods, as well as isolation and loneliness during lockdowns, which has exacerbated mental health issues. Although job security-related stress is evident among individuals in general (49) this is particularly prevalent among refugees and asylum seekers, as their limited previous experience, lack of recommendations, and inability to accredit qualifications create additional challenges (50). There is a greater concern about job insecurity in low-and middle-income countries. For example, Ozer et al. (36) in their analysis of internally displaced persons in Burkina Faso, and Palattiyil et al. (46) in their assessment of refugees in Kenya, have shown that forcibly displaced people face additional challenges in terms of food security and mobility. This literature review argues that food security challenges and dependency on host countries (camps or reception centers) exacerbate physical health problems. Additionally, refugees have limited access to public health services. For example, Syrian refugees in Jordan cannot access health facilities without providing identification documents (51). This review argues that inability to create sectoring and effective social distancing could also be attributed to physical health problems, as many refugees reside in densely populated camps with inadequate health infrastructure, making physical distancing very difficult. Additionally, the lack of access to clean water, face masks, and other personal protective equipment makes refugees more vulnerable to COVID-19.

A key challenge facing refugees is the ability to access suitable and affordable housing (52, 53). The researchers of this review argue that the pandemic has exacerbated the economic challenges that refugees face on a daily basis. Lockdowns, whether total or partial, have prevented refugees from earning their daily income, particularly since many refugees lack stable employment opportunities. It might also be difficult for refugees to obtain adequate treatment if they are infected with COVID-19 due to the decrease in their income. It is crucial to remember that whether immigrants have the legal right to work depends on their legal status, including whether they are refugees, or asylum seekers (4) For people who do not have the right to work, COVID-19 and the associated lockdown has made matters worse.

As a second research question we aimed to identify the key barriers that hamper access. This study recognizes that inadequate access to valid information is a key barrier. During the pandemic, access to digital sources was a key driver of information. According to Budak and Bostan (40) the digital gap (lack of access to computers) and social media driven misinformation (29, 46) produced major social inequalities contributing to the rise of the information access barrier. It has been shown in previous research that information asymmetry continues to affect the refugee population. According to different studies (35, 53), the most frequent sources of information include social media, traditional media, and informal networks of communication, e.g., through other refugees. Additionally, Emmer et al. (54) contend that because refugees are seen as being “in transition,” host nations seldom give access to information a high priority. Thus, rumors and misinformation may be spread and reinforced through different sources.

Healthcare workers (23, 45) acknowledge that refugees and asylum seekers lack access to resources that could enable them to receive support. A systematic review indicates several barriers such as lack of trust in the host healthcare system, communication or language barriers, and a lack of access to integrated and migration friendly services (55).

Furter, the availability of vaccinations and acceptance might not coincide (29, 35). Studies show that vaccine hesitancy may be attributed to a variety of factors; structural factors such as poverty related barriers (e.g., lack of money to buy vaccinations, to buy masks, or use transportation), difficult registration processes in the host country, language and/or communication barriers (lack of language or literacy adjusted information, poor interpretation services), digital tools demanding digital competencies, lack of health insurance, personal documentation and national bank ID’s, as well as personal barriers such as a general fear related to the effects of the COVID-19 vaccine, fear of the vaccine being religiously prohibited, and limited trust in authorities and/or the health care system (23, 29, 31, 32, 35, 41); probably reflecting prior experiences with persecution and lack of protection by authorities in the refugees home countries (56).

Given that refugees, asylum seekers, or internally displaced persons have previously suffered because of “crisis” occurrences (such as terrorism), the worry is probably not unwarranted. In the light of crisis situations, authorities may treat refugees discriminatory and brutally, including by repressing them (56). Australia (29) appear to be striving to guarantee that everyone has access to vaccinations, but they will not be successful unless they take effective steps to allay the concerns of persecution that many refugees experience.

Further, access to vaccinations and reluctance to receive them are related to refugees’ financial capacity to pay for them (31). Due to exorbitant unit prices, vaccination availability is a challenge in many poor nations, including Kenya (57). In such cases, it is crucial to take into account the host nation’s capacity to meet the requirements of both refugees and its own asylum applicants.

The factors influencing COVID-19 vaccination hesitancy among the age group (children) have not been explored; however, we hypothesize that the factors may be similar to those previously mentioned for other vulnerable populations. There is a need to further investigate these factors among children to develop targeted strategies to address vaccine hesitancy. One additional factor that may contribute to vaccination reluctance in children might be the perception of a lower risk associated with COVID-19 infection in this age group.

## Strengths and limitations

This scoping review is notable for its inclusion of asylum seekers, refugees, undocumented migrants, and internally displaced persons. In addition, this study has the advantage of adopting an explicit methodology that can be replicated to the last detail. Over the next few years, COVID-19-related research will continue to evolve, with more studies exploring similar topics. Providing specific methodology and details can enhance the search process and build upon the findings of this review.

It is imperative to acknowledge some limitations of the selected studies. The majority of studies are cross-sectional in nature, evaluating evidence at a specific point in time. The variations in evolving COVID-19 strains and shifts in vaccine policy and vaccine research can change the observed outcomes of this study. It is vital that longitudinal cohort studies are carried out to ascertain the long-term implications of the COVID-19 pandemic. Though the researchers intended to include all health and well-being-related asylum seekers, refugees, internally displaced persons, and undocumented migrants’ outcomes, targeted research on specific health issues (e.g., access to cancer healthcare and diabetes management) was not evident. It is important to acknowledge that most of the included quantitative studies have a small sample size, thus the findings should be interpreted carefully.

As part of this study, a thematic analysis approach was used to summarize the results. It would be desirable to conduct a meta-analysis to assess the epidemiological implications associated with refugee-level exposure and treatment support during the pandemic in the future.

The findings of this Review have several implications for strengthening routine public health response to the COVID-19 pandemic, as well as the needs of the asylum seekers, refugees, undocumented migrants, and internally displaced persons. COVID-19 has starkly demonstrated the imperative embedded in the notion that preventing the spread of pandemics and subsequent health and economic disruptions requires recognizing that everyone should not be left behind. Public health responses must be inclusive of all populations in order to ensure more resilient and responsive health systems for all. In the event that the health and wellbeing needs remain unmet, poorly addressed, or of low priority, the host countries will remain vulnerable.

Asylum seekers, refugees, undocumented migrants, and internally displaced persons should be involved in the development of tailored and evidence-based vaccination strategies to address specific barriers and perceptions regarding vaccinations with specific focus to COVID-19. Additionally, it will be crucial to communicate public health messages to gain the trust of the target populations and combat the spread of misinformation, as highlighted by the rollout of the COVID-19 vaccine effectively and unambiguously.

## Conclusion

As COVID-19 has shown, refugees, asylum seekers, undocumented migrants and internally displaced persons are facing a dilemma as a result of poverty, lack of support, and a pandemic. The lack of economic opportunities and housing options negatively impacts their physical health. There are a moderate to a high level of mental health challenges facing them. These challenges are further aggravated by a lack of resources, a lack of information, and a lack of adjustments in the healthcare system. It is necessary to address vaccine hesitancy in order to prevent any future outbreaks of COVID-19. There is a need for future research to identify key facilitators that can help overcome these barriers. This study includes studies focusing on refugees living in high income countries (e.g., the United Kingdom, Australia, and Italy) as well as studies from low-middle income countries (e.g., Jordan, Lebanon, Burkina Faso). As a result, the refugees, undocumented migrants, asylum seekers and internally displaced persons’ experiences can differ, and to obtain a contextually relevant picture of the state of their health and well-being during the pandemic, future literature reviews could compare evidence in high income countries and low-middle income countries.

## Author contributions

All authors listed have made a substantial, direct, and intellectual contribution to the work and approved it for publication.

## Conflict of interest

The authors declare that the research was conducted in the absence of any commercial or financial relationships that could be construed as a potential conflict of interest.

## Publisher’s note

All claims expressed in this article are solely those of the authors and do not necessarily represent those of their affiliated organizations, or those of the publisher, the editors and the reviewers. Any product that may be evaluated in this article, or claim that may be made by its manufacturer, is not guaranteed or endorsed by the publisher.
