# Supplementary material for: Health and well-being of refugees, asylum seekers, undocumented migrants, and internally displaced persons under COVID-19: a scoping review
Source: Front Public Health. 2023 Apr 26;11:1145002. doi: 10.3389/fpubh.2023.1145002 (PMC10169615; doi:10.3389/fpubh.2023.1145002)
Supplement: Supplementary file 1 [file Table_1.DOCX]

Appendix1: Glossary of migration from International Migration Law No. 34 - Glossary on Migration (20)

| Term | Meaning |
| --- | --- |
| Asylum seeker | An individual who is seeking international protection. In countries with individualized procedures, an asylum seeker is someone whose claim has not yet been finally decided on by the country in which he or she has submitted it. Not every asylum seeker will ultimately be recognized as a refugee, but every recognized refugee is initially an asylum seeker. |
| Internally displaced persons | Persons or groups of persons who have been forced or obliged to flee or to leave their homes or places of habitual residence, in particular as a result of or in order to avoid the effects of armed conflict, situations of generalized violence, violations of human rights or natural or human-made disasters, and who have not crossed an internationally recognized State border. |
| Migrant | At the international level, no universally accepted definition for “migrant” exists. The term migrant was usually understood to cover all cases where the decision to migrate was taken freely by the individual concerned for reasons of “personal convenience” and without intervention of an external compelling factor; it therefore applied to persons, and family members, moving to another country or region to better their material or social conditions and improve the prospect for themselves or their family. The United Nations defines migrant as an individual who has resided in a foreign country for more than one year irrespective of the causes, voluntary or involuntary, and the means, regular or irregular, used to migrate. Under such a definition, those travelling for shorter periods as tourists and businesspersons would not be considered migrants. However, common usage includes certain kinds of shorter-term migrants, such as seasonal farmworkers who travel for short periods to work planting or harvesting farm products |
| Refugee | A person who, “owing to a well-founded fear of persecution for reasons of race, religion, nationality, membership of a particular social group or political opinions, is outside the country of his nationality and is unable or, owing to such fear, is unwilling to avail himself of the protection of that country. |
| Undocumented migrant | A non-national who enters or stays in a country without the appropriate documentation. This includes, among others: a person (a) who has no legal documentation to enter a country but manages to enter clandestinely, (b) who enters or stays using fraudulent documentation, (c) who, after entering using legal documentation, has stayed beyond the time authorized or otherwise violated the terms of entry and remained without authorization |
