# Supplementary material for: Health and well-being of refugees, asylum seekers, undocumented migrants, and internally displaced persons under COVID-19: a scoping review
Source: Front Public Health. 2023 Apr 26;11:1145002. doi: 10.3389/fpubh.2023.1145002 (PMC10169615; doi:10.3389/fpubh.2023.1145002)
Supplement: Supplementary file 2 [file Table_2.DOCX]

# Appendix II. Summary Table

| Reference | Country | Year | Funding | Research aim/ | Design and type of intervention | Type of participants | Main findings | Limitations | Conclusion |
| --- | --- | --- | --- | --- | --- | --- | --- | --- | --- |
|  |  |  |  | objective |  |  |  |  |  |
| Budak and Bostan (2020) | Turkey | 2020 | N/A | To determine the effect of the pandemic on Syrian refugees in a single location in Turkey | Quantitative: cross-sectional survey | 414 Syrian refugees, targeted through cross-sectional survey | 30% of Syrian refugees in Turkey do not have enough pandemic information, and 45% have restricted access to PPE. | No use of inferential analysis, lack of clarity regarding the validity and reliability testing of the chosen instrument. | In order to counteract the COVID-19 outbreak, Syrian refugees should be informed about the pandemic and given protective gear. |
| Ceccon & Moscardino (2022) | Italy | 2022 | Cariparo Foundation PhD scholarship | The purpose of this study is to examine the impact of the first wave of COVID-19 and the nationwide lockdown imposed by the Italian government in spring 2020 on the mental health system | Cross-sectional study using both quantitative and qualitative methods | Young asylum seekers in Italy (n=42) | There was moderate to high satisfaction with the clarity of the communication about safety precautions among respondents, who knew the nature, source, and distribution of COVID-19; | Convenience sampling, ad-hoc use of quantitative questionnaire without validity testing, targeting only young adult. | The data indicated that asylum seekers' psychological vulnerabilities are worsened by ongoing existential uncertainty as a result of the current emergency. |
| Deal et al. (2021) | UK | 2021 | NIHR (NIHR300072) | The purpose of this study was to assess the equitable uptake of COVID-19 vaccines among undocumented migrants, asylum seekers, and refugees | Qualitative interviews | Recently arrived migrants including refugees and asylum seekers in the UK (n=32) | Out of 32 migrants, 23 (72%) were unsure whether to have the COVID-19 vaccine, and two (3%) flat-out refused. Concerns were voiced concerning the safety of the vaccines, their adverse effects, the language barrier, a lack of faith in the healthcare system, and the low level of perceived necessity. | Lack of geographical representation, limited access to interpretation services which could have increased the sample size. | In Europe and developed countries with large immigrant populations, there is a need for simple, but innovative steps to improve equitable access and uptake of the COVID-19 vaccination. |
| Gilman et al. (2020) | Greece | 2020 | Engineering and Physical Sciences Research Council | Evaluate the effectiveness of interventions to reduce the spread of COVID-19 in refugee camps | Quantitative modelling | NA | In this study, an agent-based modeling approach was used. Sectoring the camp reduced peak infection by 70% and delayed it by months. Infection rates were reduced when face masks were worn and diseased people were isolated | Individual differences and system trends, which can be difficult to replicate. | Possible interventions can decrease COVID-19's spread in refugee camps and help camp management plan interventions. |
| Guglielmi et al. (2020) | Bangladesh | 2020 | Foreign, Commonwealth & Development Office, UK | To evaluate the impact of COVID-19 on Rohingya adolescents in Bangladesh | Mixed methods study: longitudinal quantitative survey data and in-depth qualitative interviews | Rohingya asylum-seeking adolescents (n=30) | The results show that both girls and boys among the Rohingya are more likely to be at risk of food insecurity, educational and economic exclusion, and threats to their physical well-being as a result of the pandemic. | Access limitations to refugees due to lack of contact method, limitations of language interpretation. | The results of this study highlight the heightened vulnerability of teenage Rohingya girls and boys in the setting of the COVID-19 pandemic in the refugee camps of Cox's bazar. |
| Kondilis et al. (2021) | Greece | 2021 | N/A | To identify impact of the pandemic on refugees and asylum seekers | Retrospective study | Refugee and asylum seekers in Greece | Refugee and asylum seeker had a high chance of contracting COVID-19 infection. | Dependence on external datasets, poor epidemiological surveillance and tracking. | High COVID-19 transmission was found among refugees and asylum seekers in Greece vaccination rollout plans. |
| Jones et al. (2022) | Jordan | 2022 | Research and Evaluation Division of the UK Foreign Commonwealth and Development Office (FCDO) for the Gender and Adolescence: Global Evidence | To evaluate the psychosocial well-being and resilience of adolescent refugees | Mixed methods study: longitudinal quantitative survey data and in-depth qualitative interviews | 3,000 Syrian refugees, stateless Palestinians and vulnerable Jordanians | 19.3% of adolescents in the sample had moderate-to-severe depression nine months into the pandemic, with modest signs of recovery. | Use of phone-based interviews reduced opportunities to collect in-depth information. High attrition rate is another limitation. | As a result of the COVID-19 pandemic, pre-existing social inequities among refugee adolescents impacted by forced displacement have been amplified. |
|  |  |  | (GAGE) |  |  |  |  |  |  |
| Lebni et al. (2022) | Iran | 2022 | NA | To evaluate challenges in access to treatment for COVID-19 | Qualitative: semi-structured interview-based study | Afghan refugee women (n=30) | The main categories are little knowledge and information, incomplete knowledge about COVID-19, family challenges, childbirth and pregnancy problems). | Convenience sampling and lack of reflexive analysis of researcher role. | Due to their already precarious circumstances, Afghan refugee women in Iran are particularly at risk from COVID-19. |
| Liddell et al. (2021) | Australia | 2021 | Australian Research Council Linkage Project | To identify the link between COVID-19 related stressors and the mental health of refugees in Australia | Quantitative: cross-sectional survey | Refugees and asylum seekers living in Australia (n=656) | Health anxiety and PTSD were predicted by refugees' fears of contracting COVID-19 or of others contracting it. | Cross-sectional nature of study, lack of validation of research instruments in the chosen context. | The COVID-19 epidemic may have a disproportionate impact on refugees because it triggers memories of the violence and oppression they fled. |
| Lusambili et al. (2020) | Kenya | 2020 | Department of Population Health (DPH) of the Medical College at the Aga Khan University and the International Organization for Migration (UN Migration) | To evaluate access to reproductive health support during the pandemic amongst refugee women | Qualitative: in-depth interview-based study | Healthcare staff (n=25) | In the first eight months of COVID-19, the data suggest that refugee women opted more frequently for home deliveries, and healthcare providers reported seeing a decrease in service use and delays in care. | Language barriers and use of face masks made it difficult to interpret facial expressions, low sample size. | The results stress the importance of removing or reducing obstacles that impede female refugees from accessing health services. |
| Mangrio et al. (2022) | Sweden | 2022 | The Swedish Foundation for International Cooperation in Research and Education (STINT) | To evaluate barriers and facilitators to care during COVID-19 | Qualitative study: Semi-structured interviews | Health and social care workers working with refugees in Sweden | Poor public health suggestions were made for refugees, whose living situations prevented self-isolation and social distancing. Sweden's initially non-restrictive approach to the pandemic instructed health and social workers to encourage refugees to take far fewer precautions than their European neighbours.. | Convenience sampling-associated bias, interviewees gender imbalance. | Public health authorities often fail to recognise that people use varied sources of knowledge to preserve their health and that not everybody has access to healthcare and social institutions. |
| Ozer et al. (2022) | Burkina Faso | 2022 | Development Cooperation Committee of the Academy for Research and Higher Education (ARES-CCD) | To evaluate impact of COVID-19 on living and survival conditions of IDP | Qualitative study: interviews | Internally displaced persons (n=106) | 84% of the displaced people polled were not engaged in any kind of income-generating activity at all during the lockdown. | A limited number of respondents, a skewed representation of camp dwellers, and, in some cases, the inability of people to openly express their emotions all undermine the reliability of the results. | `There is a need for humanitarian support to address the well-being of internally displaced people. |
| Page et al. (2021) | USA, Switzerland, Italy and France | 2021 | National Institute of Health USA, Ministry of Education, University and Research in Italy | The purpose of this study was to characterise the initial stages of the immunisation campaign among migrants and explain why some of them were reluctant to get vaccinated against COVID-19 | Quantitative: cross-sectional survey | Undocumented migrants (n=812) | Although 86.4% of people reported that they were able to get vaccinated against COVID-19 if they wanted to, only 41.1% actually did so. | Lack of testing and validation of translated questionnaire, non-representative sample | The research revealed a discrepancy between the COVID-19 vaccine's perceived availability and its actual demand. Public health treatments targeting undocumented migrants' reluctance to get vaccinated against COVID-19 should focus on men, younger migrants, and those at low clinical risk of severe infection by using diverse communication modes to build on trust regarding vaccination in general. |
| Palattiyil et al. (2022) | Uganda | 2022 | Scottish Funding Council/Global Challenges Research Fund-Partnerships Fund | To identify the enablers and barriers to access to and use of HIV/AIDS or TB care in health systems, and at provider and individual levels | Mixed methods study: longitudinal quantitative survey data and in-depth qualitative interviews | Urban refugees (n=255) and medical practitioners, non-governmental organisations (NGOs), volunteers and village health team (VHT) members (n=8) | Access to TB or HIV/AIDS care was helped by shorter wait times, drug distribution by VHTs, close proximity to health facilities, availability of essentials like food. | Self-reporting bias, under-reporting bias, social desirability bias. | According to the results, if stakeholders are aware of the structural barriers that prevent urban refugees from receiving HIV/AIDS and TB services during pandemics like COVID-19, they will be better able to create steps to remove those barriers. |
| Salibi et al. (2022) | Lebanon | 2022 | ' 'ELRHA's Research for Health in Humanitarian Crisis (R2HC) Programme, which aims to improve health outcomes by strengthening the evidence | To evaluate vaccine acceptance | Quantitative: cross-sectional survey using four panels | Syrian refugees (n=1037) | About a third (30%) of the 1,037 recipients said they had no plans to be vaccinated. The novelty of the vaccination (35%); a desire to continue using precautionary measures (21%); a conviction that the COVID-19 vaccine is unnecessary | Low statistical power, data collection before vaccine dissemination started. | It is necessary to disseminate information that is correct, easily available, and culturally acceptable regarding the safety and efficacy of vaccines in order to address the vaccine hesitancy that exists among Syrian refugees in Lebanon. |
| Sharif-Esfahani et al. (2022) | Canada | 2022 | Social Sciences and Humanities Research Council of Canada | To evaluate the impact of the COVID-19 pandemic on Syrian refugee parents | Quantitative study: cross-sectional survey | Syrian refugee parents residing in Ontario (n=274) | 12.2% of participants reported severe depression, 26.8% reported severe anxiety, and 9.7% reported severe stress, while 24.1% of participants screened positive for PTSD. | Self-reporting, lack of confounding bias analysis, cross-sectional evaluation. | For the sake of improving the psychological health of Syrian refugee parents, government programmes should give serious consideration to addressing their fears around pandemics. |
| Shaw et al. (2022) | US | 2022 | Central New York Community Foundation, and Community Support for Refugee & Immigrant Families from Refugee Health Navigators in Syracuse, New York | To evaluate vaccination intent and behaviour amongst refugees | Quantitative: cross-sectional survey | refugee population (n=244) | From the total of 244, 140 (57.4%) were planning to get vaccinated, 43 (17.6%) were undecided, and 61 (25.0%) were against vaccination. | Low sample size, convenience sampling, selection bias. | In this research, researchers found that over a third of refugees were unwilling to get vaccinated. In addition to improved accessibility to vaccination clinics, refugees voiced a desire for more information on the advantages and safety of vaccines. |
| Thiel de Bocanegra et al. (2022) | California | 2022 | Patient-Centered Outcomes Research Institute (PCORI) Program Award (14471_UCI _IC) | To evaluate the engagement of refugee women with digital reproductive health support during the pandemic | Qualitative: semi-structured interview-based study | Community leaders and health and social service providers (n=9) and refugee women (n=27) | Whether or not refugees, particularly women, were willing to interact on virtual platforms depended on a number of factors, including demographics and personal history. The women's membership in refugee support groups in their communities aided their use of online resources. = | Lack of sample diversity, language barriers, lack of access to telephones for some refugees. | When provided in a culturally sensitive manner that takes into account refugee women's living arrangements and access to technology when they arrive in the United States, virtual groups on SRH are well received. |
| Truelove et al. (2020) | Bangladesh | 2020 | Office of Foreign Disaster Assistance (OFDA), USAID | To evaluate the impact on health and well-being and predict COVID-19 related morbidity determinants | Quantitative modelling | Refugees in Bangladesh | In 61%–92% of simulations, at least 1,000 persons were infected across all scenarios, indicating that a widespread outbreak was feasible with a single introduction of the virus into the camp. | Use of mass action model may overestimate prevalence of COVID-19 trends. | Significant healthcare capacity and infrastructure expansions may be needed to deal with a COVID-19 pandemic in a refugee settlement, which may be beyond the scope of present plans. In refugee camps around the world, it is essential to prepare for the worst. |
| Turunen et al. (2021) | Finland | 2021 | Finnish Institution for Health and Welfare, Finnish Immigration Agency, City of Espoo | To assess the prevalence of COVID-19 outbreak at a reception centre for asylum seekers | Quantitative: cross-sectional survey | Asylum seekers from different nationalities (n=260) | SARS-CoV-2 was detected in 37% of the sample, who were kept separate. Due to the high attack rate, the regional public health authorities quarantined all other clients for 14 days. Of the refugees who tested positive, 64% said they were experiencing symptoms either at the time of testing or within the previous week. | Small sample size, loss of respondents to attrition, scarcity of symptom follow-up information. | Health promotion response was more effectively coordinated thanks to the results of a voluntary mass screening. Measures to quarantine and isolate affected areas likely had a key role in stopping the spread of the disease. |
| Zhang et al. (2021) | United States | 2021 | N/A | To evaluate perceptions of the impact of COVID-19 on the health of Bhutanese and Burmese refugees | Quantitative: cross-sectional surveys | Bhutanese and Burmese refugees (n=218) | COVID-19 infection was reported by 6.9% of people. One of the most significant predictors of contracting the virus was being a female essential worker during the pandemic who also had a close relative who was infected. | Use of snowball sampling methodology restricts diversity in sample selection, use of self-reporting methods for infection tracking, only targeted refugees who can speak English, reducing population representation. | Refugees from Bhutan and Burma who worked in critical industries had a higher probability of contracting COVID-19 than those who did not. |
