# Supplementary material for: Health and well-being of refugees, asylum seekers, undocumented migrants, and internally displaced persons under COVID-19: a scoping review
Source: Front Public Health. 2023 Apr 26;11:1145002. doi: 10.3389/fpubh.2023.1145002 (PMC10169615; doi:10.3389/fpubh.2023.1145002)
Supplement: Supplementary file 3 [file Table_3.DOCX]

**Appendix III: Mixed Methods Appraisal Tool (MMAT)**

Qualitative Studies

| Reference | S1. Are there clear research questions? | S2. Do the collected data allow to address the research questions? | 1.1. Is the qualitative approach appropriate to answer the research question? | 1.2. Are the qualitative data collection methods adequate to address the research question? | 1.3. Are the findings adequately derived from the data? | 1.4. Is the interpretation of results sufficiently substantiated by data? | 1.5. Is there coherence between qualitative data sources, collection, analysis and interpretation? | Comments |
| --- | --- | --- | --- | --- | --- | --- | --- | --- |
| Deal et al. (2021) | Yes | Yes | Yes | Yes | Yes | Yes | Yes | High methodological rigour. Use of pre-interview to select participants meeting inclusion and exclusion criteria helps improve quality of participant selection and achieve thematic saturation. |
| Lebni et al. (2022) | Yes | Yes | Yes | Yes | Yes | Yes | Yes | High methodological rigour. Steps to theme identification is clear. Overall generalisability of study is high. |
| Lusambili et al. (2020) | Yes | Yes | Yes | Yes | Yes | Yes | No | Moderate methodological rigour. The qualitative findings gathered across participant groups is not well integrated. There is no clarity in the differences in type of questions posed to different participants. |
| Mangrio et al. (2022) | Yes | Yes | Yes | Yes | Yes | Yes | Yes | High methodological rigour. Clarity in data analysis and reflexive engagement of the researchers. |
| Thiel de Bocanegra et al. (2022) | Yes | Yes | Yes | Yes | Yes | Yes | Yes | High methodological rigour. The need for qualitative data is well understood. The type of questions used in the interview guide is detailed. This helps in creating replicability of research. |

Mixed Methods studies

| RefID | S1. Are there clear research questions? | S2. Do the collected data allow to address the research questions? | **5. MIXED METHODS STUDIES** | | | | |  |
| --- | --- | --- | --- | --- | --- | --- | --- | --- |
|  |  |  | 5.1. Is there an adequate rationale for using a mixed methods design to address the research question? | 5.2. Are the different components of the study effectively integrated to answer the research question? | 5.3. Are the outputs of the integration of qualitative and quantitative components adequately interpreted? | 5.4. Are divergences and inconsistencies between quantitative and qualitative results adequately addressed? | 5.5. Do the different components of the study adhere to the quality criteria of each tradition of the methods involved? | Comments |
| Ceccon and Moscardino (2022) | Yes | Yes | Yes | Yes | Yes | No | Yes | High methodological rigour. The similarities across quantitative and qualitative data is reported. However, divergences are not reported. |
| Guglielmi et al. (2020) | Yes | Yes | Yes | Yes | Yes | No | Yes | The steps taken to conduct quantitative and qualitative research is independently provided. The validity and trustworthiness of data collection instruments is high. The qualitative tool has made use of saturation of data to decide on sample size. Quantitative tool uses established measures to assess outcomes. |
| Jones et al. (2022) | Yes | Yes | Yes | Yes | Yes | No | Can't tell | The need for qualitative research as a potential follow-up to add weight to the quantitative research is recognised. There is no validation of research instruments. Overall, moderate methodological rigour. |
| Ozer et al. (17) | Yes | Yes | Yes | Yes | Yes | No | Can't tell | This research has used a mix of open and close ended questionnaire supporting both quantitative and qualitative research. However, integration of mixed methods outcomes to explain inconsistencies between the two studies is not well addressed. There is also lack of assessment of how the validity and reliability of the open and close-ended survey tool was assessed. Overall, a moderate methodological rigour is evident. |
| Palattiyil et al. (2022) | Yes | Yes | Yes | No | Yes | No | Yes | Moderate methodological rigour. The divergencies between quantitative and qualitative data is not reported. The qualitative data interpretation and analysis does not provide links to quantitative outcomes. |
|  |  |  |  |  |  |  |  |  |

Quantitative Descriptive Studies

| RefID | S1. Are there clear research questions? | S2. Do the collected data allow to address the research questions? | **4. QUANTITATIVE DESCRIPTIVE STUDIES** | | | | |  |
| --- | --- | --- | --- | --- | --- | --- | --- | --- |
|  |  |  | 4.1. Is the sampling strategy relevant to address the research question? | 4.2. Is the sample representative of the target population? | 4.3. Are the measurements appropriate? | 4.4. Is the risk of nonresponse bias low? | 4.5. Is the statistical analysis appropriate to answer the research question? | Comments |
| Budak and Bostan (2020) | Yes | Yes | Yes | Yes | Yes | Can't tell | Yes | Moderate methodological rigour. The rationale behind decision on sample size is not clearly defined. No efforts made to minimise non-response bias. |
| Kurt et al. (2021) | Yes | Yes | Yes | Yes | Yes | Yes | Yes | High methodological rigour. The sampling could have been improved to look for refugees through platforms other than social media. |
| Liddell et al. (2021b) | Yes | Yes | Yes | Yes | Yes | No | Yes | High methodological rigour. Clarity in instruments used. Validity and internal consistency declared. However, participant selection and response bias minimisation is not characterised. |
| Lidell et al. (2021a) | Yes | Yes | Yes | Yes | Yes | Yes | Yes | High methodological rigour. Use of validated measures and the right statistical tools to answer research questions. |
| Page et al. (2022) | Yes | Yes | Yes | Yes | Yes | Yes | Yes | High methodological rigour. The study used several strategies to reduce the risk of recruitment and measurement bias by addressing the main barriers limiting undocumented 'migrants' participations in health programmes such as fear of personal data misuse and sociocultural factors. |
| Salibi et al. (2021a) | Yes | Yes | Yes | Yes | Yes | No | Yes | High methodological rigour. Longitudinal wave of data used can help reduce participant non-response bias. |
| Salibi et al. (2021b) | Yes | Yes | Yes | Yes | Yes | Can't tell | Yes | High methodological rigour. |
| Sharif-Esfahani et al. (2022) | Yes | Yes | Yes | Yes | Yes | Can't tell | Yes | High methodological rigour. Internal consistency and reliability of instruments used is declared. Lack of clarity on how non-response bias is addressed. |
| Shaw et al.,(2022) | Yes | Yes | Yes | Yes | Yes | Yes | Yes | High methodological rigour. Participant follow-up and selection clearly detailed. Participants loss to follow-up highlighted reducing bias of reporting. |
| Turunen et al. (2021) | Yes | Yes | Yes | Yes | Yes | Yes | Yes | High methodological rigour. Participant follow-up helped reduce loss of participants through the course of the study. |
| Zhang et al. (2021) | Yes | Yes | Yes | Yes | Yes | Can't Tell | Yes | High methodological rigour. Good justification of chosen statistical model and analysis. |

Non-Randomised Studies

| RefID |  |  | **3. NON-RANDOMIZED STUDIES** | | | | |  |
| --- | --- | --- | --- | --- | --- | --- | --- | --- |
|  | S1. Are there clear research questions? | S2. Do the collected data allow to address the research questions? | 3.1. Are the participants representative of the target population? | 3.2. Are measurements appropriate regarding both the outcome and intervention (or exposure)? | 3.3. Are there complete outcome data? | 3.4. Are the confounders accounted for in the design and analysis? | 3.5. During the study period, is the intervention administered (or exposure occurred) as intended? | Comments |
| Gilman et al. (2020) | Yes | Yes | Yes | Yes | Yes | Can't tell | Can't tell | Moderate methodological rigour. Assumptions made through modelling and associated limitations of choice of modelling are not effectively discussed. |
| Kondilis et al. (2021) | Yes | Yes | Yes | Yes | Yes | Yes | Yes | High methodological rigour. The study contributes to epidemiological surveillance data in a specific location. |
| Truelove et al. (2020) | Yes | Yes | Yes | Yes | Yes | Can't tell | Can't tell | Moderate methodological rigour. Assumptions made through modelling and associated limitations of choice of modelling are not effectively discussed. |
